# How to treat VAP due to MDR pathogens in ICU patients

**DOI:** 10.1186/1471-2334-14-135

**Published:** 2014-11-28

**Authors:** José Garnacho-Montero, Yael Corcia-Palomo, Rosario Amaya-Villar, Luis Martin-Villen

**Affiliations:** Unidad Clínica de Cuidados Críticos y Urgencias, Hospital Universitario Virgen del Rocío, Sevilla, Spain; Instituto de Biomedicina de Sevilla (IBiS), Hospital Universitario Virgen del Rocío/CSIC/Universidad de Sevilla, Spain; Red Española de Investigación en Patología Infecciosa (REIPI), Hospital Universitario Virgen del Rocío, Sevilla, Spain

## Abstract

**Background:**

The increasing occurrence of multidrug resistant (MDR) bacteria arises at a time when there is a lack of antibiotics active against these pathogens and few new antimicrobials are in the pipelines of the pharmaceutical industry. Treatment of ventilator-associated pneumonia (VAP) caused especially by MDR Gram-negative bacilli (GNB) represents a real challenge due to the dearth of treatment options.

**Methods:**

We searched the medical literature relevant about management of ventilator-associated pneumonia caused by multi-drug resistant pathogens including GNB and methicillin-resistant *S. aureus*.

**Results:**

Empirical therapy should be prescribed based on the local pattern of susceptibilities. Colistin and tigecycline are in many cases the unique options for the treatment of many episodes of VAP caused by MDR-GNB. Tigecyline (not licensed for treatment of pneumonia) should be used with an initial bolus of 200 mg followed by 100 mg every 12 h. The need for a loading dose and the administration of high doses of colistin (9 million IU/day in two or three doses) is currently accepted. Vancomycin has been considered the treatment of choice for pneumonia due to MRSA although linezolid may provide higher rate of clinical cure for MRSA VAP with a good safety profile. The initial antibiotic treatment must be reassessed and simplify in accordance of culture results.

**Conclusions:**

Empirical treatment of VAP due to MDR pathogens should be based on knowledge of local ecology. A strategy combining early high doses of effective agents with subsequent simplification in the light of microbiologic information is recommended.

## Introduction

Ventilator-associated pneumonia (VAP) is one of the most dreaded nosocomial infection. VAP appears to have a low attributable mortality although episodes caused by multi-drug resistant (MDR) pathogens is associated with a significant attributable mortality [1, 2]. Potential MDR pathogens include: *P. aeruginosa*, *Acinetobacter* spp, extended spectrum beta-lactamase (ESBL)-producing Enterobacteriaceae, *Klebsiella*-producing carbapanamase strains, *Stenotrophomonas maltophilia*, and methicillin-resistant *S. aureus* (MRSA).

In critically ill patients, the susceptibility of the bacteria isolated in a VAP depends on the duration of stay in the ICU and on mechanical ventilation as well as the previous use of antibiotics [3]. VAP has been conventionally classified into early and late onset. Early-onset VAP occurs within less than 96 hours of ICU admission and is generally due to antimicrobial-sensitive bacteria. Late-onset VAP occurs after 96 hours of ICU admission and may be caused by MDR pathogens. Other risk factors of MDR pathogens include prior antibiotic use within the preceding 90 days, frequency of antibiotic resistance in the community or hospital, and the immunocompromised state [4]. However, different studies have described a high rate of VAP due to MDR pathogens in episodes occurring in the first days of mechanical ventilation highlighting the importance of local ecology [5–7].

Diagnosing of VAP is difficult because it requires a thorough assessment of clinical data, radiological findings, and microbiological results. There are no foolproof tools to determine whether the patient has a VAP. When the clinical suspicion of VAP is high, empirical antimicrobial therapy must be initiated promptly because both delayed and inadequate treatments have been associated with increased rate of morbidity and mortality [4]. Nevertheless, one third of the patients with VAP only exhibit clinical criteria of sepsis [8]. In patients with no signs of severe sepsis or septic shock and no organisms present on Gram’s staining, antimicrobial therapy can be withheld pending culture results [9, 10].

Current guidelines recommend empirical coverage of Gram-negative bacilli (GNB) with a third or forth generation cephalosporin, piperacillin-tazobactam or a carbapenem in combination with a fluoroquinolone or an aminoglycoside [11]. However, the problem arises when a high proportion of the GNB are resistant to these antibiotics. One of the consequences of a greater prevalence of antimicrobial resistance is an increased recognition of inadequate antimicrobial treatment of infection. Few alternatives are available for treatment of these multi-drug resistant GNB.

### Antibiotics for MDR-GNB

#### Carbapenems

Carbapenems have constituted the mainstay of VAP for many years. Imipenem, meropenem and doripenem have similar spectrum although doripenem is the most active carbapenem against *P aeruginosa*[12]. No carbapenem seems to be superior to another in clinical trials that have evaluated these agents in late-onset VAP [13, 14] or in VAP caused by *P aeruginosa*[15]. Optimization of carbapenem dosages (extended infusion of meropenem or doripenem) achieves improved pharmacokinetic and pharmacodynamic target attainments and is associated with higher rate of clinical cure in VAP [16, 17]. Dual-carbapenem therapy (ertapenem plus meropenem or doripenem) against KPC-producing *K. pneumoniae* has been evaluated in animal models [18]. The beneficial effect may be related to the KPC enzyme’s preferential affinity for ertapenem hindering doripenem or meropenem degradation and allowing the action of this carbapenem. A successful recovery with the use of this combination in a patient with bacteremic VAP due to pan-resistant KPC-producing *K. pneumoniae* has been recently reported [19].

#### Colistin

Polymyxins are a group of polypeptide cationic antibiotics. Only polymyxin B and polymyxin E (colistin) are used in clinical practice. Colistin acts in a way that does not promote cross-resistance and is unlikely to be associated with swift selection of resistant strains. Nowadays, colistin is the antimicrobial with the greatest level of in vitro activity against multi-drug resistant GNB including *A baumannii*, *P aeruginosa*, extended spectrum beta-lactamase (ESBL)-producing Enterobacteriaceae or *Klebsiella*-producing carbapanamase strains. However, some GNB such as *Proteus* spp, *Providencia* spp, *Morganella morganii* and *Serratia marcescens* are resistant [20].

Over the last decade, our knowledge on the clinical pharmacokinetic of colistin has increased considerably. Several studies have pointed out that intravenous administration of colistimethate sodium (CMS) may lead to suboptimal plasma concentrations and is associated with higher mortality. Thus, low daily dosage of i.v. colistin has been identified as an independent predictor of mortality (OR 0.81, 95% CI 0.68-0.96) in patients with VAP [21]. In 13 critically ill patients with VAP caused by GNB, colistin was undetectable in BAL performed at 2 h after the start of the CMS infusion (2 million IU 8-hourly) [22].

The need for a loading dose and the administration of high doses of colistin has been recently demonstrated. Plachouras et al [23] described in 18 critically ill patients receiving 3 million IU of CMS 8-hourly that plasma colistin concentrations were sub-optimal for 2-3 days before reaching steady state. The authors recommended a loading dose of 9 million IU and 4.5 million IU 12-hourly because colistin displayed a half-life that was significantly long in relation to the dosing interval. The clinical efficacy and the lack of toxicity of this regimen have been confirmed in a series of critically ill patients with bacteremia or VAP caused by MDR-GNB [24]. In contrast, a loading dose of 6 MU may be adequate [25], and our preliminary results indicate that a loading dose of 4.5 UI of colistin may be sufficient to reach therapeutic levels in non-obese critically ill patients with MDR-VAP [26].

The efficacy of colistin in VAP caused by MDR-GNB has been demonstrated in several retrospective and prospective series [27–30]. These studies have evaluated colistin mostly in the directed therapy being scarce the information about the use of this antibiotic empirically. A multicenter randomized clinical trial (RCT) that is currently ongoing has been designed to assess the efficacy and safety of colistin compared to meropenem in the empirical therapy of VAP with high suspicion of MDR-GNB [31]. While awaiting the results of this RCT, the empirical use of colistin may justify in institutions where there is a high rate of infections due to MDR-GNB [32].

Synergistic activity of colistin and rifampicin combination against different GNB has been documented in experimental models [33, 34]. Nevertheless, a recent RCT failed to demonstrate clinical superiority of the combination of colistin plus rifampicin over monotherapy with colistin in patients with severe infections (two-thirds with VAP) caused by extensively drug-resistant *Acinetobacter baumannii*[35]. The results were identical in another clinical trial that compared colistin plus rifampicin with colistin in patients with VAP caused by carbapenem-resistant *Acinetobacter baumannii*[36].

Different *in vitro* studies have documented the existence of a potent synergism of the combination of colistin with a glycopeptide against carbapenem-resistant *A baumannii*[37, 38]. In a retrospective series, we did not find clinical benefit of this combination in patients with *A baumannii* VAP and observed a higher rate of renal failure in comparison with monotherapy with colistin [39]. Conversely, a multicenter study that included a heterogeneous group of infections caused by different GNBs concluded that therapy with colistin plus a glycopeptide ≥ 5 days was a protective factor for 30-day mortality [40].

#### Tigecycline

Tigecycline is a broad spectrum antibiotic with potent *in vitro* activity against anaerobic and aerobic Gram-positive bacteria and Gram-negative pathogens with the exceptions of *P aeruginosa* and *Proteus* ssp. Tigecycline is active against *A. baumannii*, including some colistin-resistant strains [41].

Notwithstanding, tigecycline is not licensed for treatment of hospital-acquired pneumonia, including VAP. In a multicenter, randomized, double-blind, study that compared 50 mg every 12 h (loading dose 100 mg) with imipenem, tigecycline was inferior to the imipenem/cilastatin regimen for the clinically evaluable population. This difference appeared to have been caused by results in VAP patients in whom there was a higher mortality in the tigecycline group that did not reach statistical significance [42]. Nevertheless, small series and observational studies have reported acceptable results with the use of tigecycline for MDR-VAP [43, 44]. Very recently, in a matched cohort analysis adjusted for propensity score, episodes of *A baumannii* VAP treated with tigecycline had a statistically significant excess of mortality in comparison to patients treated with colistin. The excess mortality of tigecycline was significant only among those with MIC >2 μg/mL, but not for those with MIC ≦ 2 μg/mL. All isolates were susceptible to colistin [45].

Dose of tigeclycline approved for intra-abdominal and skin and soft tissue infections (50 mg every 12 h with a loading dose of 100 mg) does not achieve adequate concentrations for pulmonary infections [46]. A recent randomized phase 2 trial has evaluated the clinical efficacy of two high-dosage tigecycline regimens versus imipenem-cilastatin for treatment of hospital-acquired pneumonia. Although the number of patients included is too low to draw conclusions in terms of mortality, 200 mg followed by 100 mg every 12 h achieved the greatest rate of clinical cure [47]. Therefore, it seems necessary to administer, in an episode with suspicion of extremely resistant GNB, this high dose in order to avoid insufficient levels in lung tissue and, at least empirically, always in combination therapy (a carbapenem or colistin are probably the best options).

#### Fosfomycin

Fosfomycin is an “old” antimicrobial that shows potent bactericidal action against many Gram-negative and Gram-positive pathogens. The drug shows little toxicity. Regrettably, resistance develops rapidly when fosfomycin is used as monotherapy. Since fosfomycin shows synergistic action with other antimicrobials it is an interesting option to treat a wide range of infections, including VAP caused by MDR pathogens [48].

This antibiotic has gained recently relevance for the treatment of extensively resistant GNB especially *K pneumonia* or *P aeruginosa* due to the lack of valid alternatives. A recent series has reported an acceptable response in patients with VAP treated with high doses (24 g/day) of fosfomycin always in combination with other antimicrobials [49].

### Antibiotics for resistant Gram-positive cocci

Empirical coverage of MRSA is less troublesome. Vancomycin has been considered the treatment of choice for pneumonia due to MRSA [4]. Almost all MRSA isolates are susceptible to vancomycin and only anecdotal cases of MRSA resistant to this glycopeptide have been reported. However, an interesting issue to keep in mind is the MIC of MRSA to vancomycin. Several studies in patients with MRSA pneumonia or bacteremia (mainly from lung source) have observed a higher rate of treatment failure and mortality in episodes with MIC ≥ 1.5 mg/L treated with vancomycin [50, 51]. Lung infection has been identified as an independent predictor of treatment failure in MRSA bacteremia treated with vancomycin and not explained by MIC [52], perhaps reflecting the poor penetration of this antibiotic into the lung tissue [53].

Current guidelines recommend a loading dose of 25-30 mg/kg followed by vancomycin dosages of 15-20 mg/kg given every 8-12 hours for patients with normal renal function [54]. Monitoring of trough concentrations is necessary after the forth dose to maintain serum trough concentrations of 15-20 mg/L increase efficacy and improve clinical outcomes. An AUC to MIC ratio ≥400 has been shown to predict more favorable microbiological and clinical outcomes in cases of *S. aureus* pneumonia [55].

Teicoplanin might be an acceptable alternative to treat pneumonia caused by MRSA. However, the administration of high teicoplanin doses (12 mg/kg teicoplanin every 12 h the first 2 days followed by 12 mg/kg once daily) is needed to reach sufficient antibiotic concentrations in lung tissues at steady state [56]. We do not recommend teicoplanin for VAP due to the uncertainties about the correct doses, impossibility of level measurements, and the availability of alternatives such as vancomycin or linezolid.

Linezolid has been shown to have a better pharmacokinetic profile than vancomycin [57]. The *post hoc* analysis of two randomized, double-blind studies concluded that linezolid therapy was associated with significantly better clinical cure and survival rates than therapy with vancomycin in the subgroup of patients with MRSA VAP [58]. In a recent double-blind, controlled trial, linezolid, compared with vancomycin, achieved a higher clinical and microbiological response rate (the latter was not statistically significant) despite vancomycin dose optimization, together with a lower incidence of all types of renal adverse effects in patients with MRSA pneumonia. However, mortality was unaffected [59]. Limitations of this study include imbalanced distribution of medical comorbidities and the number of patients excluded to reach the per-protocol group.

Although tigecycline is active against MRSA, clinical cure of cases of MRSA was lower with tigecycline than with the comparator in the clinical trial that evaluated this antibiotic in hospital-acquired pneumonia [42]. Therefore, we do not advocate the use of tigecycline for MRSA pneumonia and a specific antibiotic against this Gram-positive bacterium is necessary.

Ceftaroline is a cephalosporin with broad gram-positive activity, including MRSA. Its gram-negative activity includes common respiratory pathogens and members of the Enterobacteriaceae. However, ceftaroline is currently only approved for acute bacterial skin infections and community-acquired pneumonia. This new cephalosporin has a promising role in the treatment of VAP but clinical data are not currently available. It should be used in combination with another antimicrobial to cover GNB such as *P aeruginosa* or ESBL-producing Enterobacteriaceae.

### Nebulized antibiotics

The administration of nebulized antibiotics has been proposed to provide high levels of the drug in the lung and to reduce the systemic toxicity associated with intravenous antibiotics. The concentration in the respiratory secretion of the nebulized antibiotic may be 20 to 100 fold greater than the *in-vitro* MIC of the organisms being treated [60]. Large randomized trials are needed to define the impact on clinical outcomes of nebulized antibiotics as adjunctive therapy in MDR-VAP.

Although diverse antibiotics have been nebulized, the most extensive experience exists with aminoglycosides and colistin. The use of appropriate devices is essential to assure clinical and microbiological utility of nebulized antibiotics. During mechanical ventilation, high amounts of the particles dispersed by conventional nebulizers remain in the ventilatory circuits and the tracheobronchial tree and, therefore, less drug is available in the alveolar compartment.

The use of aerosolized colistin for multi-drug resistant GNB pneumonia increases cure rates and may be reasonably efficacious and safe [61]. The use of inhaled colistin was independently associated with clinical cure in a retrospective study (OR 2.53, 95% CI 1.11-5.76) although mortality was unaffected. Nevertheless, other studies have concluded that the use of aerosolized colistin in conjunction with intravenous colistin did not provide additional therapeutic benefit to patients with MDR VAP due to gram-negative bacteria [62]. Moreover, a randomized controlled trial of nebulized CMS as adjunctive therapy of ventilator-associated pneumonia caused by GNB failed to demonstrate beneficial effect on clinical outcome [63]. Regarding aminoglycosides, several studies have evaluated tobramycin or amikacin with promising results [64, 65].

### Duration of treatment

There is no consensus regarding the duration of antibiotic treatment for patients with VAP due to MDR. In a randomized clinical trial that included patients with VAP and adequate empirical antimicrobial therapy, short-course (8 days) treatment of VAP has no difference in terms of mortality compared to long-course (15 days) treatment. In VAP caused by non-fermenting gram-negative bacilli including *P aeruginosa*, rate of recurrence was significantly higher (40.6% *vs* 25.4%) in patients receiving 8 days of treatment [66]. A recent meta-analysis of four RCTs also concluded that a short-course of antibiotic may be enough to treat VAP although the issue of length of therapy in MDR VAP has not been specifically evaluated [67]. A prospective study showed that a procalcitonin-based strategy (recommending that physicians stop antibiotics when the procalcitonin concentration was <0.5 ng/mL, or had decreased by ≥80%) did not negatively influence outcomes although the subgroup of patients with MDR-VAP was not specifically assessed [68].

## Conclusions

Empirical treatment of a VAP with a high probability of a MDR pathogen is one of the major challenges facing an intensivist. Similarly, it is complex the directed therapy especially when a very-difficult-to-treat MDR-GNB (i.e. carbapenemase-producing *K. pneumoniae,* extremely drug-resistant *A baumannii* or *P aeruginosa*) is confirmed as the etiology of the pneumonia. Our personal point of view for the treatment of VAP caused by MDR pathogens is summarized as a Decalogue in Table 1. Doses of antibiotics recommended for these infections are depicted in Table 2.

**Table 1:** **Decalogue to treat VAP caused by MRD pathogens**

1	Antimicrobials used in the empirical regimens should be chosen based on the local pattern of susceptibility.
2	Initiation of antimicrobial therapy should not be delayed in patients with high probability of VAP especially if the infection originates severe sepsis or septic shock.
3	In patients with no signs of severe sepsis or septic shock and no organisms present on Gram’s staining, antimicrobial therapy can be withheld pending culture results.
4	When a high rate of episodes is caused by extremely resistant GNB, empirical use of colistin and/or tygecycline may be justified.
5	The inclusion of a carbapenem (in extended infusion) in this empirical therapy seems reasonable especially for pathogens not covered by these antibiotics.
6	Addition of vancomycin or linezolid is recommended is Units with high prevalence of MRSA (>10% of episodes caused by MRSA).
7	The initial antibiotic treatment must be reassessed when the culture results are available. Depending on the clinical progress and the microbiological findings, clinicians should adjust therapy accordingly.
8	In episodes caused by very-difficult-to-treat GNB, it seems prudent to maintain combination therapy (if possible) until the clinical course appears clearly favorable.
9	Nebulized antibiotics should be considered in the directed therapy of patients who are nonresponsive to systemic antibiotics or in episodes caused by GNB strains with high CMI (intermediate).
10	Not all patients with MDR-VAP have to be treated for two weeks. Courses of treatment should be individualized. Procalcitonin may be of aid to stop antibiotics after eight days of adequate antimicrobial therapy.

**Table 2:** **Recommended doses of antimicrobials use in VAP caused by MDR pathogens in patients with normal renal function**

Antibiotic	Loading dose	Daily dose	Observations
Imipenem^*^	Not required	1 g/6-8 h	Extended or prolonged infusion is not possible due to drug instability
Meropenem^*^	Not required	1-2 g/8 h	Extended infusion (3-4 hours) is recommended.
Doripenem^*^	Not required	500 mg-1 g/8 h	Extended infusion (3-4 hours) is recommended.
Colistin^*^	4.5-9 UI	9 UI/day in 2 or 3 dose	Loading dose is necessary.
Tigecycline	200 mg	100 mg/ 12 h	Without approval by regulatory agencies.
Fosfomycin^*^	Not required	24 g/day (in four doses)	Always in combination therapy.
Vancomycin^*^	25-30 mg/kg (based on ABW)	15-20 mg/kg (based on ABW) every 8-12 hours	Monitor trough concentrations after the forth dose; serum trough levels of 15-20 mg/L for MRSA VAP.
Linezolid	Not required	600 mg/ 12 h	It should be changed to vancomycin in the directed therapy of patients with good clinical evolution and *S aureus* with vancomycin MIC ≤ 1 mg/L.

*Dose adjustment is necessary in case of renal dysfunction.
